# Fire rather than nitrogen addition affects understory plant communities in the short term in a coniferous‐broadleaf mixed forest

**DOI:** 10.1002/ece3.4263

**Published:** 2018-07-22

**Authors:** Mengjun Hu, Yanchun Liu, Zhaolin Sun, Kesheng Zhang, Yinzhan Liu, Renhui Miao, Shiqiang Wan

**Affiliations:** ^1^ International Joint Research Laboratory for Global Change Ecology School of Life Sciences Henan University Kaifeng Henan China; ^2^ Luoyang Institute of Science and Technology Luoyang Henan China

**Keywords:** burning, climate transitional zone, disturbance, light availability, recovery

## Abstract

Increasing fire risk and atmospheric nitrogen (N) deposition have the potential to alter plant community structure and composition, with consequent impacts on biodiversity and ecosystem functioning. This study was conducted to examine short‐term responses of understory plant community to burning and N addition in a coniferous‐broadleaved mixed forest of the subtropical‐temperate transition zone in Central China. The experiment used a pair‐nested design, with four treatments (control, burning, N addition, and burning plus N addition) and five replicates. Species richness, cover, and density of woody and herbaceous plants were monitored for 3 years after a low‐severity fire in the spring of 2014. Burning, but not N addition, significantly stimulated the cover (+15.2%, absolute change) and density (+62.8%) of woody species as well as herb richness (+1.2 species/m^2^, absolute change), cover (+25.5%, absolute change), and density (+602.4%) across the seven sampling dates from June 2014 to October 2016. Light availability, soil temperature, and prefire community composition could be primarily responsible for the understory community recovery after the low‐severity fire. The observations suggest that light availability and soil temperature are more important than nutrients in structuring understory plant community in the mixed forest of the subtropical‐temperate transition zone in Central China. Legacy woody and herb species dominated the understory vegetation over the 3 years after fire, indicating strong resistance and resilience of forest understory plant community and biodiversity to abrupt environmental perturbation.

## 1 INTRODUCTION

At the global scale, total area burned each year has increased from 301 million ha in 1997 to 377 million ha in 2011 (Giglio, Randerson & Werf, 2013). The frequency and intensity of fire are also expected to increase in forests under future warmer and drier climate scenarios (Abatzoglou & Williams, 2016; Pechony & Shindell, 2010; Schoennagel et al., 2017). Fires could have profound impacts on forest structure, community composition, and ecosystem function (Fultz et al., 2016; Holland, Clarke & Bennett, 2017; Pellegrini et al., 2017; Wan, Hui & Luo, 2001).

Understory plant communities are an important component in forests and contribute substantially to biodiversity and ecosystem functions, such as wildlife habitat, dominant species regeneration, soil retention, and nutrient cycling (Barbier, Gosselin & Balandier, 2008; Roberts, 2004; Zhang et al., 2015a). In addition, understory plant species are more sensitive to disturbance than canopy trees, serving as a critical indicator of future community composition and ecosystem health (Gilliam, 2007; Meier, Bratton & Duffy, 1995). Fire has been widely found to increase the density, cover, and richness of understory species with time since fire (Abella & Springer, 2015; Metlen & Fiedler, 2006; Webster & Halpern, 2010). For example, the cover of graminoids maintains a consistent increase throughout the 10 postfire years in ponderosa pine forests of the United States (Abella & Fornwalt, 2015). The changes have been attributed to the resistance and resilience of forest understory community to burning (Abella & Fornwalt, 2015; Metlen & Fiedler, 2006). However, the impacts of fire on understory vegetation recovery are highly variable because of differences in region (Abella & Springer, 2015) and forest type (e.g., coniferous and broadleaf; Veldman, Mattingly & Brudvig, 2013) as well as severity, frequency, and season of fires (Crotteau, Varner & Ritchie, 2013; Knapp, Schwilk, Kane & Keeley, 2007; Veldman et al., 2013). Fire can affect understory vegetation recovery in two different ways. On the one hand, fire may directly combust aboveground vegetation and litter layer and stimulate seed germination and resprouting ability (Clarke et al., 2013). For example, rapid recover of understory species density after burning has been observed in second‐growth forests of the United States due to the regrowth of resprouting shrub species and rhizomatous colonization, as well as herbs with short life longevity (Phillips & Waldrop, 2008). On the other hand, fire may indirectly affect seedling growth by altering light availability, soil physical, chemical, and biological properties (Gundale et al., 2005; Hiers, O'Brien, Will & Mitchell, 2007; North, Oakley, Fiegener, Gray & Barbour, 2005). Hence, distinguishing the direct and indirect effects of fire on the regeneration dynamics of understory vegetation will facilitate the mechanistic understanding of forest community structure and composition under increasing global fire risk.

Fire can reduce fuel nitrogen (N) amount, but instantaneously increase soil N availability although enhancing soil hydrophobicity and N mineralization (Shakesby & Doerr, 2006; Wan et al., 2001), with consequent influences on plant growth in forest ecosystems (Aber et al., 2001). In addition to burning, increased soil N availability under atmospheric N deposition can also stimulate plant growth and aboveground biomass in forests (Pregitzer, Burton, Zak & Talhelm, 2008; Thomas, Canham, Weathers & Goodale, 2010). Fast‐growing nitrophilous plants under elevated soil N availability in the short term may reduce coexistence of other species (Bobbink et al., 2010; Gilliam, 2006; Lu, Mo, Gilliam, Zhou & Fang, 2010), leading to long‐term species loss in forests and tundra. For example, N enrichment can elevate cover of vascular plants and reduce cover of bryophytes in tundra ecosystems (Bobbink et al., 2010). Chronical ammonium and nitrate inputs have been well demonstrated to result in reductions of understory species diversity in temperate and boreal forests which are commonly N limited (Bobbink et al., 2010; Gilliam, 2006; Strengbom, Nordin, Näsholm & Ericson, 2001). Given that both fire and N deposition can elevate soil N availability, we hypothesize that burning and N addition could synergistically influence community composition and diversity of understory plants in forests.

A field experiment with burning and N addition was established in May 2014 in a coniferous‐broadleaf mixed forest in Central China. The forest is located in a transitional zone from subtropical to warm temperate region where plant growth and ecosystem functions are considered to be sensitive to global change (Zhang, Dong, Fu & Wu, 2003). Over the past half century (1964–2014), mean annual temperature in the local area increased from 14.7°C in 1964 to 17.7°C in 2014 (China Meteorological Data Sharing Service System, http://data.cma.gov.cn). Low‐severity fire caused by human activities is widespread in this region (Su, He & Chen, 2015). Evidence also shows that the transitional zone is experiencing atmosphere N deposition (Liu et al., 2013). Many studies have examined responses of soil microbial communities (Carson & Zeglin, 2018; Liu, Xu, Hong & Wan, 2010) and plant photosynthesis (Zhang, Niu, Xu & Han, 2008) to burning and N addition. However, there are few studies focusing on the impacts of burning and N addition on forest community structure and composition (Hurteau & North, 2008), especially in transition zone. The objectives of this study were to address: (a) how understory plant community structure and composition respond to burning and N addition; (b) and did burning and N addition interactively impact on understory plant community.

## 2 MATERIALS AND METHODS

### 2.1 Study site

The study site is located at the Mountain Xian (32°06′N, 114°01′E, 204 m a.s.l.) of Nanwan Forest Service, Xinyang, Henan, China. Long‐term mean annual precipitation (1951–2014) is approximately 1,063 mm with 66% occurring from May to September. Mean annual temperature is 15.2°C with monthly mean temperature ranging from 1.9°C in January to 33.6°C in July (China Meteorological Data Sharing Service System, http://data.cma.gov.cn). The soil at the study site is classified as a Haplic Luvisols (FAO classification). Mean soil bulk density, litter depth, and pH are 0.99 g/cm^3^, 1.2 cm, and 4.5, respectively. The dominant canopy species in the mixed forest are German oak (*Quercus acutissima* Carruth.) and Masson pine (*Pinus massoniana* Lamb.). The understory shrub species are primarily by *Vitex negundo* L., *Lindera glauca* (Sieb. et Zucc.) BI, *Rubus corchorifolius* L. f., and *Symplocos chinensis* (Lour.) Druce. The herb layers are dominated by perennial grass *Carex rigescens* (Franch.) and *Lygodium japonicum* (Thunb.) Sw. The stand is secondary forest with an age of about 25 years. Mean height of canopy trees, shrubs, and herbs are 8, 1.3, and 0.18 m, respectively.

### 2.2 Experimental design

The experimental site was located on a south‐facing hillslope (approximately 20% in slope) with the altitude ranging from 193 to 224 m. The west side of the hillslope was burned accidentally by a ground fire with the flame height of 1.6–2.2 m on 15 April 2014. Fire‐tolerant species, including *V. negundo*,* L. glauca*,* S. chinensis*,* Dalbergia hupean*, and *Q. acutissima*, survived after the fire. However, the east side was left intact due to the existence of a small trail between the two sides. The experimental area had not been burned for at least 20 years when fire records started, and had uniform plant composition and site characteristics. The fire consumed all the litter layer and most aboveground parts of the understory shrubs and herbs, leading to 30% mortality of canopy trees with scorch height of 1.7–2.5 m. Consequently, the fire was subject to low‐severity fire according to the depth (2.5 ± 0.4 cm) of the burned organic soil (Kasischke et al., 2008).

**Figure 1:**
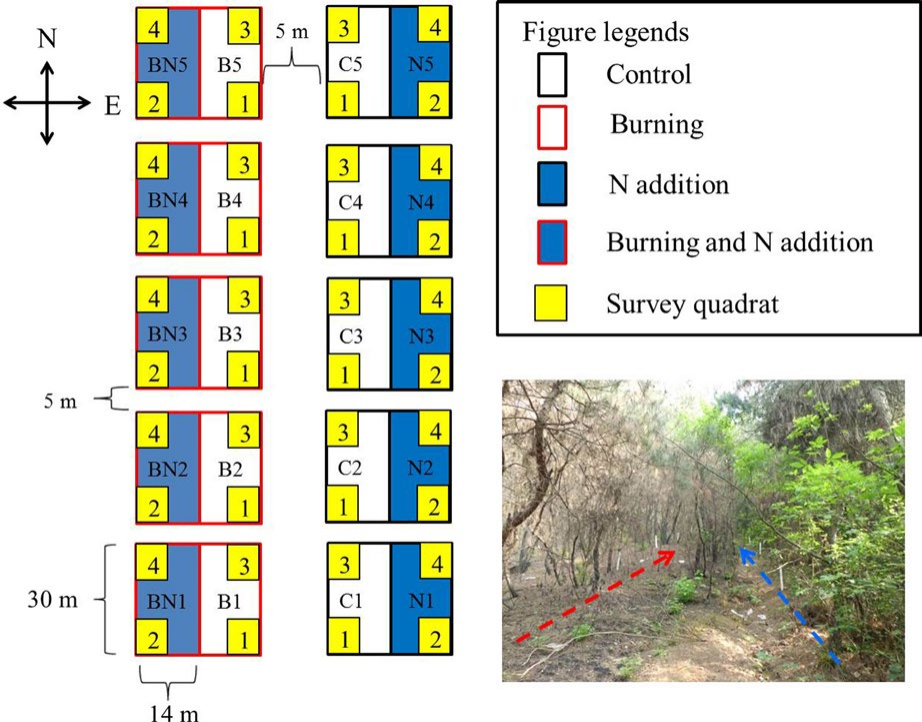
Plot layout of the experiment

The experiment used a pair‐nested design with five replicates. Five pairs of 30 m × 30 m plots were set up along the hillslope 2 weeks after the fire. In each pair, the plot in the west side was assigned as the burning treatment and the other one in the east site as the unburned treatment. The distance between the paired plots in both the burned and unburned sides is approximately 5 m. Each plot was divided into two 14 m × 30 m subplots with 2 m distance between the subplots. The two subplots were assigned to N addition and control treatments. In order to avoid the slope impacts on plots via surface runoff, the N addition subplot in each plot was arranged in the west and east sides in the burned and unburned area (Figure 1). Thus, there were five replicates for each treatment [control (C), burning (B), N addition (N), and burning plus N addition (BN)]. Each N addition subplot received 50 kg N ha^−1^ year^−1^ in the form of urea from 2014 to 2016. Nitrogen was applied once on 23 July 2014 and three times (16.67 kg N ha^−1^) in May, July, and September in both 2015 and 2016. The N addition level was chosen according to the background rate of N deposition in this region and a N‐fertilization experiment in the same region (Zhang et al., 2015b).

The field surveys were performed on 15 June 2014, October 2014, May 2015, October 2015, May 2016, August 2016, and October 2016. The understory plants were divided into woody and herbaceous species. Woody species (<5 cm stem diameter at breast height, including tree seedlings, shrubs, and vines) were monitored within the two permanent 5 m × 5 m quadrats in each subplot, and a 1 m × 1 m herbaceous subquadrat was set up in each quadrat. The species richness of woody and herbaceous plants was recorded as the occurrence of the number of plant species in each quadrat and subquadrat, respectively. Cover of woody species was visually estimated for all species in each quadrat. The herb cover was estimated visually using a square grid method (Yang et al., 2012). The number of individuals (stems) for all woody and herbaceous species was counted to calculate the density of woody and herbaceous species in each quadrat and subquadrat, respectively. We also calculated the relative density of resprouters (the persistence of adult plants, which regenerate from protected buds in aboveground or basal stems; Clarke et al., 2013) and species (stems within single species contribute to total stems for all species). In addition, given the two plots in each pair were close to each other, it is reasonable to assume similar species and density of understory plants between the two plots. Therefore, understory woody and herbaceous species in the burned plots were classified as either “legacy” (from the prefire community) occurring in the control plots or “new” species (postfire colonizing) if they are absent in the control plots (Abella & Fornwalt, 2015). To evaluate how the understory community response to fire, we classified plant species based on family, functional groups, life history, photostability, regeneration, and legacy/new status (Supporting Information Table S1).

Photosynthetic available radiation (PAR) was measured with a Li‐Cor Quantum Sensor (Li‐6400, Li‐Cor Inc., Lincoln, NE, USA) 1.3 m above forest floor in each quadrat, corresponding to the mean height of woody plants. The measurements were conducted between 08:00 and 11:00 on a clear day in October 2015, May, August, and October 2016. Thirty points were recorded within each quadrat.

Three soil cores were collected at the depth of 0–10 cm in each subplot with a soil auger (5 cm in diameter and 10 cm in depth) in May 2015, October 2015, May 2016, August 2016, and October 2016. The three soil cores were mixed together, sieved with a 2‐mm mesh to separate the roots and stones, and then stored at 4°C before analysis. Soil available N (the sum of NH_4_^+^‐N and NO_3_^−^‐N) was extracted from 10 g fresh soil with 50 ml 2 M KCl and measured by Discrete Auto Analyzer (SmartChem 200, WestCo Scientific Instruments Inc., Italy).

Soil temperature at the depth of 10 cm was measured three times per month in each subplot using a thermocouple probe (Li‐8100‐201) attached to the Li‐8100. Volumetric soil water content (0–10 cm) was measured at the same time with the measurement of soil temperature in 2015 and 2016 using a portable Time Domain Reflectometer (TDR) equipment (Soil moisture equipment Corp., Santa Barbara, CA, USA).

### 2.3 Data analysis

Repeated‐measures ANOVAs were used to examine the effects of sampling date, burning, N addition, and their interactions. Between‐subject effects were evaluated as burning or N addition treatment and within‐subject effects were sampling date. Three‐way ANOVAs were used to examine the effects of block, burning, N addition, and their possible interactions on the variables in each sampling date. Multiple comparisons were used to examine the differences in sampling date of legacy species or new species after burning. Effects of block were tested together with the treatments in all the above analyses, but they were not discussed in this study. All dates were log‐transformed to meet normality and homogeneity assumptions of ANOVA. Simple linear analyses were used to examine relationships of understory plant variables with understory PAR and soil temperature. All statistical analyses were conducted with SAS V.8.1 software (SAS Institute, Cary, NC).

## 3 RESULTS

### 3.1 Light, soil available N, soil temperature, and water content

**Figure 2:**
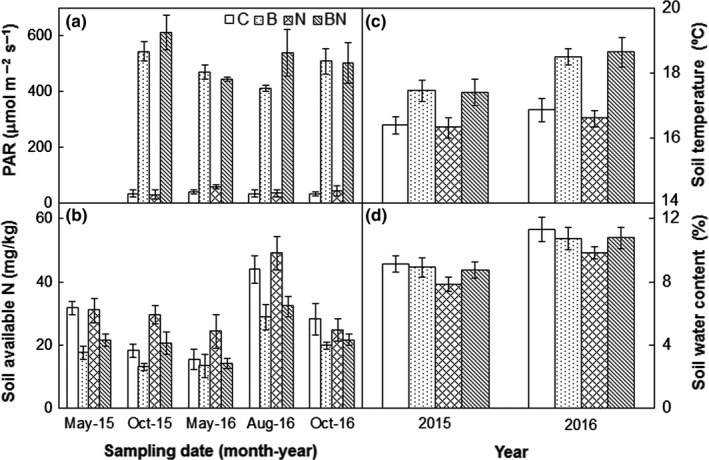
Effects of burning and N addition on understory photosynthetic active radiation (PAR) (from October 2015 to October 2016), soil available N (the sum of NH_4_^+^‐N and NO_3_^−^‐N) at the depth of 10 cm (from May 2015 to October 2016), soil temperature and water content from 2015 to 2016 (mean ± *SE*). C: control; B: burning; N: N addition; BN: burning plus N addition

Burning significantly improved understory PAR, on average, by 12.1‐fold (*p *<* *0.001, Table 1) across all the four sampling dates of October 2015, May, August, and October 2016 (Figure 2a). The positive effects of burning on understory PAR also varied with sampling date (*p *<* *0.001). Neither N addition nor its interactions with burning affected understory PAR (both *p *>* *0.05, Table 1). Burning decreased soil available N by 31.7%, whereas N addition stimulated it by 16.6% averaged from May 2015 to October 2016 (Figure 2b). However, the treatment effects on soil available N did not vary with sampling date (all *p *>* *0.05, Table 1). Across the 2 years, burning significantly stimulated soil temperature by 1.5°C (*p *<* *0.001, Figure 2c). The positive effects of burning on soil temperature also varied with year (*p *<* *0.001, Table 1). There was a significant block effect on soil temperature (*p *<* *0.001). Neither N addition nor its interactions with burning affected soil temperature (both *p *>* *0.05). No effects of burning, N addition, or their interactions on soil water content were observed (all *p *>* *0.05, Table 1; Figure 2d).

**Table 1:** Results (*F*‐values) of repeated‐measure ANOVAs with a pair‐nest design on the effects of block, burning (B), N addition (N), date, and their potential interactions on understory photosynthetic active radiation (PAR), soil available N (available N), soil temperature (ST), and water content (SM)

	PAR	Available N	ST	SM
Block	0.99	4.75	23.35***	2.72
B	374.71***	53.47***	131.6***	0.26
N	0.97	8.92*	0.21	2.33
B × N	0.50	0.07	0.92	2.00
Date	2.46	32.96***	97.44***	271.89***
Date × Block	1.45	1.83	1.11	3.58*
Date × B	3.63*	2.37	24.78***	0.40
Date × N	1.05	1.76	0.00	0.02
Date × B × N	1.70	0.96	1.57	0.73

*Note*.

*p <* *0.05; ***p <* *0.001.

### 3.2 Richness, cover, and density of woody species

**Figure 3:**
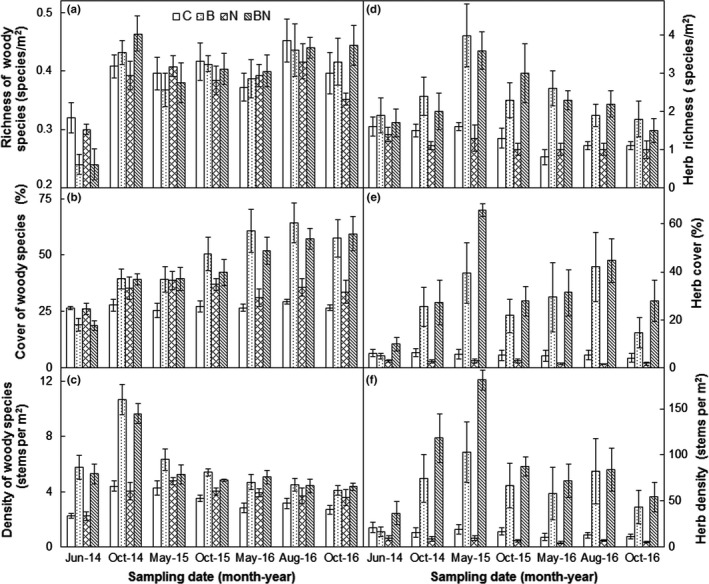
Effects of burning and N addition on the richness (a), cover (b), and density (c) of woody species as well as herb richness (d), cover (e), and density (f) from June 2014 to October 2016 (mean ± *SE*). C: control; B: burning; N: N addition; BN: burning plus N addition

No effects of burning, N addition, or their interactions on the richness of woody species were observed (all *p *>* *0.05, Table 2). However, burning interacted with sampling date to affect it (*p *<* *0.001). Burning significantly suppressed the richness of woody species by 22.6% in June 2014, but did not affect it in the following six sampling dates (Figure 3a and Supporting Information Figure S1a). Burning elevated the cover of woody species, on average, by 15.2% (absolute change, *p* < 0.001) over the 3 years. In addition, the burning effects also changed with sampling date (*p* < 0.001, Table 2). Burning suppressed the cover of woody species by 7.5% in June 2014, but increased it in other six sampling dates (Figure 3b). The burning‐induced enhancements on the cover of woody species increased dramatically from 7.8% in October 2014 to 27.5% in May 2016 and then kept relatively constant in the third year (Figure 3b and Supporting Information Figure S1b). No effects of N addition or its interaction with burning and/or sampling date on the cover of woody species were found (all *p *> 0.05, Table 2).

**Table 2:** Results (*F*‐values) of repeated‐measure ANOVAs with a pair‐nest design on the effects of block, burning (B), N addition (N), date, and their potential interactions on the richness, cover, and density of woody species, and the relative density of resprouters, as well as herb richness, cover, density, and the relative density of perennial herbs

	Woody plants				Herbs			
	Richness	Coverage	Density	Resprouters	Density	Coverage	Richness	Perennial
Block	0.63	1.42	0.96	0.96	1.24	0.50	1.09	1.18
B	0.05	16.17**	56.05***	51.53***	30.57***	25.66***	19.78***	6.54*
N	0.02	0.25	0.02	3.22	0.79	0.26	0.24	0.26
B × N	0.58	1.79	1.94	0.22	2.13	1.22	0.03	0.12
Date	27.26***	24.19***	28.85***	20.34***	11.79***	9.28***	7.98***	0.15
Date × Block	1.95*	0.74	1.15	0.8	0.87	0.72	0.74	0.82
Date × B	4.54***	17.68***	15.85***	10.64***	11.06***	9.44***	5.67***	0.68
Date × N	0.52	0.98	1.20	1.7	1.33	0.91	0.6	1.07
Date × B × N	0.46	0.88	0.17	0.4	1.54	0.80	0.71	0.61

*Note*.

*p <* *0.05; **p <* *0.01; ***p <* *0.001.

Across the 3 years, burning significantly stimulated the density of woody species by 62.8% (*p *< 0.001, Figure 3c and Supporting Information Figure S1c), whereas neither N addition nor its interactions with burning affected it (both *p *>* *0.05, Table 2). The burning effects on the density of woody species changed with sampling date (*p* < 0.001). The density of woody species was significantly enhanced by 1.4‐fold in the first year (147.5% in June 2014 and 141.1% in October 2014). However, the burning‐induced stimulations of the density of woody species were much lower and relatively consistent in the second (28.0% and 35.9% in May and October 2015) and third years (44.0%, 30.2%, and 35.4% in May, August, and October 2016; Figure 3c and Supporting Information Figure S1c).

### 3.3 Herb richness, cover, and density

Burning stimulated herb richness, on average, by 1.2 species/m^2^ (absolute change, *p *< 0.001, Table 2) over the seven sampling dates (Figure 3d and Supporting Information Figure S2a). The enhancements of herb richness were insignificant 2 months after fire (0.3 species/m^2^ in June 2014), increased up to 0.9 species/m^2^ in the late growing season of the first year (October 2014), continued to increase in the second year (2.4 species/m^2^ and 1.5 species/m^2^ in May and October 2015), peaked at the early growing season (1.6 species/m^2^ in May 2016), and then declined in the middle (1.0 species/m^2^ in August 2016) and late growing season (0.6 species/m^2^ in October 2016) of the third year (Figure 3d and Supporting Information Figure S2a). No effects of N addition or its interaction with burning and/or sampling date were found on herb richness (all *p *> 0.05, Table 2).

Across the seven sampling dates, burning significantly enhanced herb cover and density, on average, by 25.5% (absolute change, *p *<* *0.001) and 602.4% (*p *<* *0.001, Table 2), respectively. In addition, the burning effects on these two variables changed with time (both *p *<* *0.001). Irrespective of its neutral effect on herb cover and density in June 2014 burning significantly stimulated herb cover and density in other six sampling dates (all *p *<* *0.01, Figure 3e,f, Supporting information Figure S2b,c). Moreover, the increases in herb cover and density were generally higher in the spring (May) and summer (August) than autumn (October). Nitrogen addition or its interaction with burning and/or sampling date had no effects on these two herb parameters (all *p *>* *0.05, Table 2).

### 3.4 Persistence and colonization of species

**Figure 4:**
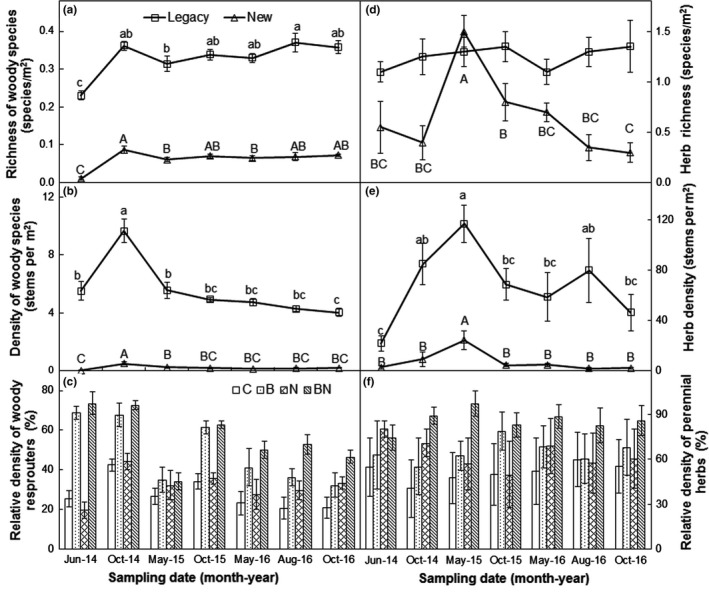
Richness (a) and density (b) of woody species as well as richness (d) and density (e) of legacy and new herb species in the burned plots from June 2014 to October 2016 (b). Relative density of resprouters (c) and perennial herbs (f) in response to burning and/or N addition. Letters compare means (*p *<* *0.05) separately for legacy species (a, b, c) and new species (A, B, C) among years. C: control; B: burning; N: N addition; BN: burning plus N addition

Woody species composition showed strong resistance and resilience to the low‐severity fire (Figure 4a–c). Across the 3 years after fire, the richness of legacy woody species was 7.5 times higher than that of new colonizers. The richness of legacy (0.23 species/m^2^) and new woody species (0.01 species/m^2^) was low right after fire in June 2014 (Figure 4a), but increased up to 0.36 species/m^2^ and 0.09 species/m^2^ in October 2014, respectively, and keep relatively consistent in the second and third years. Across the 3 years, the density of legacy woody species was 51.5 times higher than that of new species under burning (Figure 4b). The density of legacy and new woody species increased from June 2014 (5.51 and 0.03 stems/m^2^) to October 2014 (9.67 and 0.49 stems/m^2^) and then declined in the second and third years (Figure 4b). Burning significantly stimulated the relative density of resprouters by 22.9% (absolute change, *p *<* *0.001, Figure 4c), whereas neither N addition nor its interactions with burning affected it across the seven sampling dates (both *p *>* *0.05, Table 2). The burning effects on the relative density of resprouters changed with sampling date (*p* < 0.001). The burning‐induced stimulations of the relative density of resprouters were higher in the first year (37.8%) than the second (16.2%) and third years (17.3%; Figure 4c).

The richness of legacy and new herb species showed differential responses to burning across the 3 years. For example, the richness of legacy herb species exhibited relative stability after burning over the 3 years (Figure 4d). However, the richness of new herb species increased from the first year (0.55 and 0.40 species/m^2^ in June and October 2014) to the second year (1.50 and 0.80 species/m^2^ in May and October 2015), but declined in the third year (0.70, 0.35, and 0.30 species/m^2^ in May, August, and October 2016). Across the 3 years, the density of legacy herb species was 17.7 times higher than that of new species under burning. There was strong temporal variability in the density of legacy herb species after burning, with the lowest value (21.85 stems/m^2^) in June 2014 and the highest (117.05 stems/m^2^) in May 2015 (Figure 4e). The density of new herb species also varied over the 3 years, increased from June 2014 (2.60 stems/m^2^) to May 2015 (24.25 stems/m^2^) and then declined over time (Figure 4e). Perennial herbs were a dominant part of the postfire community over the 3 years (Figure 4f). Burning elevated the relative density of perennial herbs, on average, by 18.1% (absolute change, *p* < 0.001) over the 3 years. No effects of N addition or its interaction with burning and/or sampling date on the relative density of perennial herbs were found (all *p *> 0.05, Table 2).

### 3.5 Relative density of dominant species

**Figure 5:**
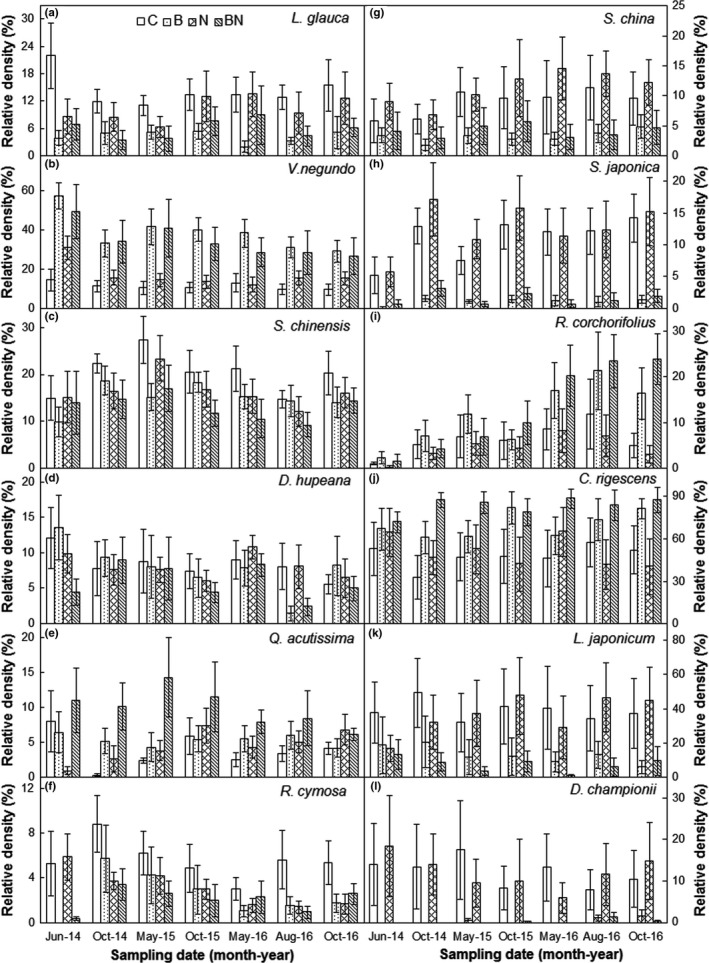
Effects of burning and N addition on the relative density of dominant woody species (*L.g*., *V.n*., *S.c*., *D.h*., *Q.a*., *R.c*., *S.c.h*., *S.j*., *R.c*.) and herb species (*C.r*.; *L.j*., *D.c*.) from June 2014 to October 2016 (mean ± *SE*). C: control; B: burning; N: N addition; BN: burning plus N addition. *L.g*., *Lindera glauca*;* V.n*., *Vitex negundo*;* S.c*., *Symplocos chinensis*;* D.h*., *Dalbergia hupean*;* Q.a*., *Quercus acutissima; R.c*., *Rosa cymosa*;* S.c.h*., *Smilax china*;* S.j*., *Serissa japonica*;* R.c*., *Rubus corchorifolius*;* C.r*., *Carex rigescens*;* L.j*., *Lygodium japonicum*;* D.c*., *Dryopteris championii*

The response patterns of dominant individual species were idiosyncratic across the seven sampling dates (Figure 5). Burning significantly stimulated the relative density of *V. negundo* within woody species and *C. rigescens* within herb species by 22.5% (absolute change, *p *<* *0.01, Figure 5b) and 27.2% (*p *<* *0.05, Figure 5j), but suppressed the relative density of *L. glauca* (woody), *Serissa japonica* (woody), and *Dryopteris championii* (herb) by 7.2% (*p *<* *0.05, Figure 5a), 10.5% (*p *<* *0.001, Figure 5h), and 11.7% (*p *<* *0.05, Figure 5l) across the seven sampling dates, respectively. The burning effects on the relative density of *V. negundo*,* S. japonica*, and *Rubus corchorifolius* (woody) changed with sampling date (all *p *<* *0.01, Table 3). No effects of N addition or its interaction with burning and/or sampling date on the relative density of species were found (all *p *>* *0.05). There was a significant block effect on *S. chinensis* (*p *<* *0.05).

**Table 3:** Results (*F*‐values) of repeated‐measure ANOVAs with a pair‐nest design on the effects of block, burning (B), N addition (N), date, and their potential interactions on the relative density of dominant species

	Woody plants									Herbs		
	*L.g*.	*V.n*.	*S.c*.	*D.h*.	*Q.a*.	*R.c*.	*S.c.h*.	*S.j*.	*R.c*.	*C.r*.	*L.j*.	*D.c*.
Block	2.20	1.15	3.94*	2.00	1.09	1.13	0.73	1.88	2.3	1.19	0.98	0.86
B	7.45*	12.46**	3.03	0.43	3.37	3.27	4.00	19.68***	4.61	5.58*	4.33	5.02*
N	0.19	0.01	1.37	0.30	1.88	1.68	0.26	0.19	0.01	0.48	0.10	0.00
B × N	1.13	0.59	0.12	0.15	1.08	0.83	0.05	0.06	0.23	0.24	0.01	0.00
Date	1.72	18.52***	6.57***	3.37**	1.04	3.66**	2.99*	12.38***	18.94***	0.39	0.56	0.65
Date × Block	0.77	1.64	1.99*	0.82	0.59	0.96	1.14	1.00	1.52	0.71	0.42	1.30
Date × B	0.60	4.16**	1.39	1.58	1.38	1.54	1.82	6.18***	6.6***	1.1	0.87	0.79
Date × N	1.44	1.39	1.66	1.27	1.17	1.24	0.36	0.93	0.83	1.48	0.98	0.71
Date × B × N	1.12	1.52	0.62	0.54	1.34	0.50	0.49	0.43	0.95	0.35	0.53	0.73

*Notes*.

*L.g*., *Lindera glauca*;* V.n*., *Vitex negundo*;* S.c*., *Symplocos chinensis*;* D.h*., *Dalbergia hupean*;* Q.a*., *Quercus acutissima*;* R.c*., *Rosa cymosa*;* S.c.h*., *Smilax china*;* S.j*., *Serissa japonica*;* R.c*., *Rubus corchorifolius*;* C.r*., *Carex rigescens*;* L.j*., *Lygodium japonicum*;* D.c*., *Dryopteris championii*.

*p < 0.05; **p < 0.01; ***p < 0.001.

### 3.6 Control factors over understory plant community

**Figure 6:**
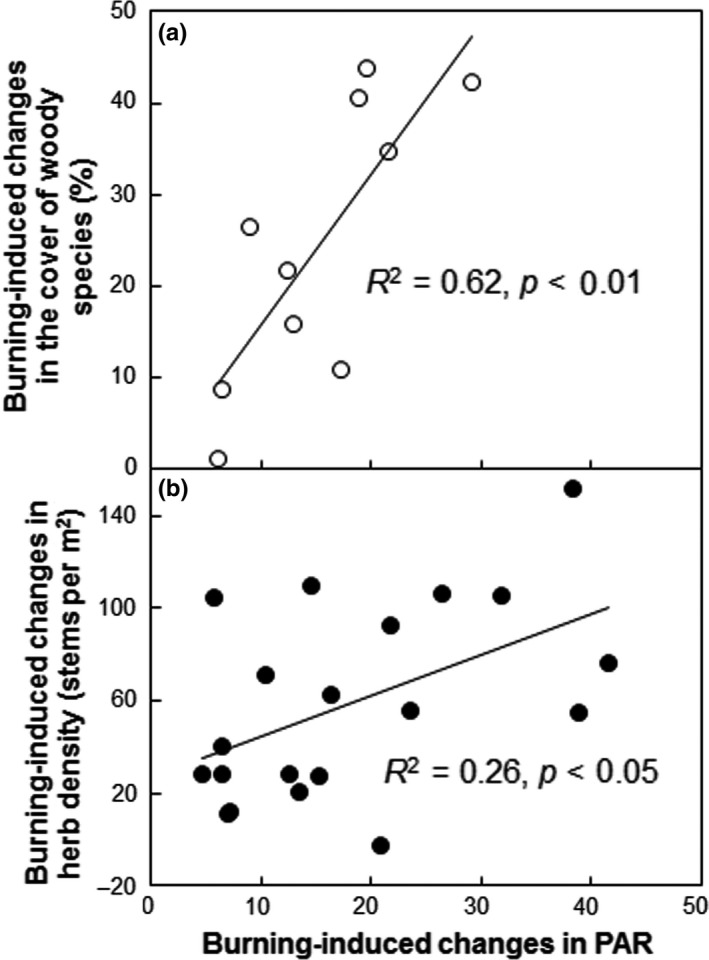
Dependence of burning‐induced changes in the cover of woody species (a) and herb density (b) on the changes in understory PAR. Each data point (open circles) represents the difference of the mean value between the burned and unburned subplots in each pair. Each data point (filled circles) represents the difference between the burned and unburned plots in each pair and sampling date from October 2015 to October 2016

**Figure 7:**
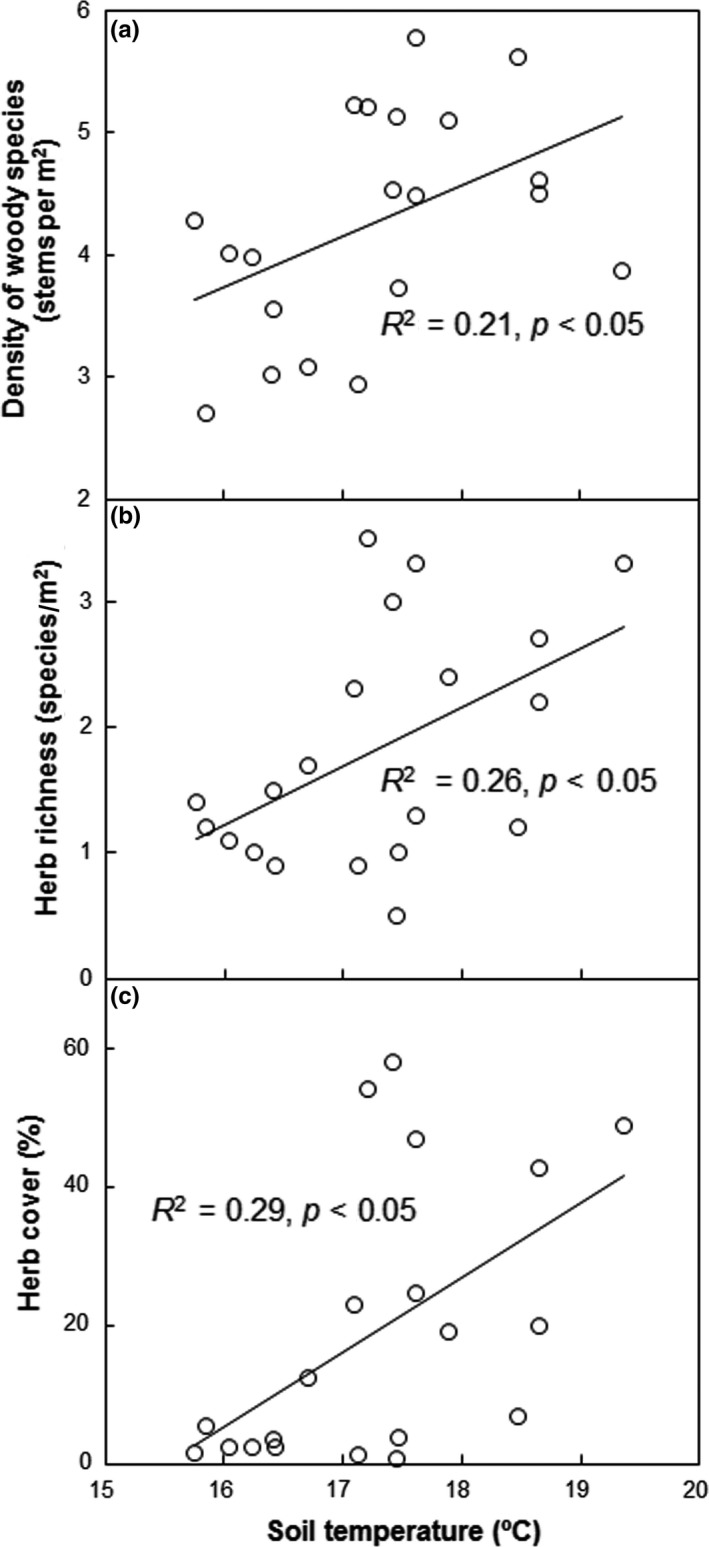
Relationship between the density of woody species (a), herb richness (b) and cover (c), and soil temperature. Each data point represents the mean value in one subplot

The absolute changes in the cover of woody species showed a positively linear dependence upon the relative changes in PAR under burning over the four sampling dates (*p *<* *0.01, Figure 6a). Similar to the cover of woody species, the absolute changes in herb density also positively correlated with the relative changes in PAR caused by burning (*p *<* *0.05) across the four sampling dates from October 2015 to October 2016 when PAR was monitored (Figure 6b). Across all the treatment from 2015 to 2016, the density of woody species, herb richness, and herb cover increased linearly with soil temperature (Figure 7a–c).

## 4 DISCUSSION

In this study, we observed a rapid recovery of species richness, cover, and density of understory woody and herbaceous plants over the 3 years after a low‐severity fire. No effects of N addition on the understory species were detected over the first 3 years. Both light availability and soil temperature were predominant factors influencing understory community recovery. In addition, burning had marginal effects on woody species composition, but increased new colonizers for herb layer. Woody and herbaceous plants were dominated by resprouters and perennial herbs after burning, respectively. Therefore, prefire community composition should be considered in models of postfire recovery of forest understory vegetation, which could help to predict forest vegetation dynamics under abrupt environmental perturbation.

### 4.1 Burning effect on understory plants

In the coniferous‐broadleaf mixed forest in Central China, burning only suppressed the richness of woody species right after fire, whereas notably enhanced herb richness, cover, and density over the first 3 years. Our observations are consistent with those reported in a *Quercus Pyrenaica* forest (Calvo, Tárrega & Luis, 1991), an old‐growth ponderosa pine forest (Laughlin et al., 2004), and some mixed forests (Huisinga, Laughlin, Fulé, Springer & McGlone, 2005; Knapp et al., 2007), but contrast with the results of some other studies that the richness, cover, and/or density of understory plants need relatively longer time to recover after burning (Abella & Fornwalt, 2015; Abella & Springer, 2015; Metlen & Fiedler, 2006; Webster & Halpern, 2010). Fire severity influences postfire recovery of understory vegetation (Keeley, 2009). Given lower depth of the burned organic soil, loss of understory woody species, and tree mortality observed, it is reasonable to conclude that this fire could be a low‐severity fire. Understory vegetation could rapidly recover after the low‐severity fire due to the resprouting ability of remnant canopy trees and shrubs as well as germination from soil seed bank (Crotteau et al., 2013; Phillips & Waldrop, 2008). In addition, light resource also influences forest vegetation cover or richness (Barbier et al., 2008). Improved light availability can stimulate woody cover and herb density at our experiment site (Figure 6). Fire can increase solar radiation at the soil surface, resulting in elevated soil temperatures from 2015 to 2016 (Figure 2c). Soil temperature plays an important role in breaking seed dormancy and promoting seed germination (Santana et al., 2010). In our study, the observation that soil temperature significantly contributed to the stimulation in the density of woody species, herb richness, and herb cover across all treatments and years (Figure 7a–c) suggests temperature‐mediated response of understory vegetation recovery to fire. Enhanced plant root growth and reduced litter depth after burning may also stimulate herb richness (Huisinga et al., 2005; Laughlin et al., 2004; North et al., 2005; Phillips & Waldrop, 2008). Reduced litterfall production after burning in 2015 (−38.0%, *p *<* *0.01) partially supported the above argument.

Reduced richness and cover of woody species 2 months after fire in the coniferous‐broadleaf mixed forest observed in this study are consistent with the widely demonstrated declines in understory plant cover in the first year after fire in various forests (Abella & Fornwalt, 2015; Metlen & Fiedler, 2006), which could be explained by the consumption of aboveground tissue by fire (Abella & Fornwalt, 2015). However, the initial reductions in the cover of woody species were reversed in the summer of the first year due to the little impacts of fire on their roots and rapid regeneration and growth (Supporting Information Figure S3). The increasing trend of cover enhancements of woody species in subsequent sampling dates could be largely attributable to the improved light condition after fire (Figure 6a). The reductions in canopy tree density were exacerbated with year (Supporting Information Figure S4), whereas the improvement of light availability kept relatively consistent over time. Consequently, woody plants tended to maximize the utilization of light resources and showed an adaptive strategy by increasing individual size but reducing individual number (density) in the second and third years (Wright, 2002).

In our study, the burning‐induced increments in the density of woody species declined with year after fire could have been explained by the population dynamic of resprouting species (the persistence of adult plants, which regenerate vegetatively from protected buds) after fire (Enright, Marsula, Lamont & Wissel, 1998). The dramatic increments in the relative density of resprouters 2 months after fire and the declining stimulation over the subsequent sampling dates supported the above argument (Figure 4c). *V. negundo* (shrub) was a dominant part of the resprouters and favored the low‐severity fire at our experiment site (Figure 5b). Moreover, the dominance richness of legacy woody species, in combination with the repaid recovery of the density of woody species, suggests strong resistance and resilience of understory woody community composition to fire disturbance.

The enhancement in herb richness in this coniferous‐broadleaf mixed forest increased right after fire, peaked in the spring of 2015, then declined over time, which could have been largely ascribed to new colonizers in the burned area (Abella & Fornwalt, 2015). The new colonizers germinated strongly in the spring of 2015, which partially supported the above argument (Figure 4d). In addition, the greater increment in the cover of woody species in the second and third years may cause light limitation on herb layer (Supporting Information Figure S5; Bloom & Mallik, 2004), thus leading to declined response of herb richness in the third year. The observations suggest that the low‐severity fire benefit to an influx of new colonizers, leading to changes in herb community composition.

The higher increments in herb cover and density in the spring and summer than autumn could have been ascribed to the differences in plant phenology induced by burning. Burning could advance the pre‐anthesis stage, delay the senescence of reproductive parts forbs (Wrobleski & Kauffman, 2003), and stimulate seed germination in the spring and plant size in the summer. In addition, burning increased the relative density of *C. rigescens*, contributing to the dominance of the perennial herb (Figure 5j). Burning may advance leaf senescence of *C. rigescens* (personal observation) under burning (Supporting Information Figure S6), thus leading to relatively lower increments in herb cover and density in the autumn. Moreover, burning‐induced changes in herb community composition also affect herb cover and density. Greater density of legacy species that dominate the herb layer in the spring of 2015 supported the above argument (Figure 4e).

The differential dynamic responses of woody and herbaceous species to burning observed in this mixed forest could be explained by different strategies for resource use, competitive ability, and physiological traits (Lu, Ma, Zhang & Fu, 2006). Compared with woody plants, herbs generally have shorter longevity and shallower roots (Knoop & Walker, 1985), thus are more sensitive to the disturbance and respond faster than woody plants.

### 4.2 Effect of N addition on understory plants

Soil N availability is the major limiting nutrient for plant growth in forests ecosystems and plant species are adapted to soil with low‐N availability (Aerts & Chapin, 1999). Given the increases in plant aboveground biomass following N addition (Bobbink et al., 2010) and negative correlations between herb richness and soil available N (Lu et al., 2010), it is expected that N addition may decrease understory plant richness. However, no changes in the richness of woody or herbaceous species under N addition were detected over the first 3 years. The observations were in line with the previous studies in a mixed‐conifer forest (Hurteau & North, 2008) and a large‐scale meta‐analysis across North America and Europe (De Schrijver et al., 2011), but contrast with the results of some other studies that chronic N addition can decrease understory species richness in temperate and boreal forest ecosystems (Bobbink et al., 2010; Gilliam, 2006). The neutral effects of N addition on understory species richness may be attributable to the shorter durations of N addition. The N addition was conducted for only 3 years, and thus, cumulative effects of N deposition may need long‐term monitoring to detect (Bobbink et al., 2010). Ecosystem background N deposition and the level of N addition have been well demonstrated to influence the responses of understory species richness to N deposition (Lu et al., 2010). The experimental N addition (50 kg N ha^−1^ year^−1^) increased soil N availability at our experimental site under the relatively high background rate of N deposition (20–25 kg N ha^−1^ year^−1^). However, fine root biomass was not affected by N addition (Supporting Information Figure S3), probably due to the occurrence of N saturation in this forest (Tian, Wang, Sun & Niu, 2016). In contrast, the positive effects of N addition on leaf N content of the three dominant shrub species observed in this study suggest that plant growth is partially constrained by soil N availability in this region (Supporting Information Figure S7). Therefore, long‐term research is essential to fully understand the realistic responses of the transitional zone from subtropical to warm temperate region under global N enrichment. Moreover, little effects of N addition on understory vegetation recovery after burning observed in this study indicate that other abiotic factors such as light, water, and some essential nutrients (Hurteau & North, 2008; Lu et al., 2010) may be more important than soil N availability in regulating understory plant growth after fire in the short term.

In conclusion, the rapid recovery of understory woody and herbaceous plants after fire in this climate transitional forest could have been attributed to the enhancements in light availability and soil temperature induced by burning as well as prefire community composition. The dominance of *V. negundo* (shrub) and *C. rigescens* (perennial herb) after fire demonstrated that *V. negundo* and *C. rigescens* rather than other species adapt to the low‐severity fire. No effects of N addition on the understory species over the first 3 years indicate the adaptation of understory plants to soil with the relatively high background rate of N deposition and that light resource and soil temperature are more important than nutrient resource in regulating understory plant community in the short‐term scale. The short‐term responses of understory woody and herbaceous community to natural and/or anthropogenic disturbance may contribute to the biodiversity and ecosystem function in the long term in the coniferous‐broadleaf mixed forest of the transitional zone from subtropical to warm temperate region.

## CONFLICT OF INTEREST

None declared.

## AUTHOR CONTRIBUTIONS

Shiqiang Wan conceived the ideas and designed methodology; Mengjun Hu, Yanchun Liu, Zhaolin Sun, Kesheng Zhang, Yinzhan Liu, Renhui Miao collected the data; Mengjun Hu and Shiqiang Wan analyzed the data; Mengjun Hu and Shiqiang Wan led the writing of the manuscript. All authors contributed critically to the drafts and gave final approval for publication.

## DATA ACCESSIBILITY

All data used in this study are included in the manuscript and supporting information.
