# Supplementary material for: Fire rather than nitrogen addition affects understory plant communities in the short term in a coniferous‐broadleaf mixed forest
Source: Ecol Evol. 2018 Jul 22;8(16):8135–48. doi: 10.1002/ece3.4263 (PMC6144994; doi:10.1002/ece3.4263)

SUPPLEMENTARY DATA

TABLE S1. Functional groups, life history, plant traits, and legacy/new status of the species found in the experimental plots from 2014 to 2016.

Species	Family	Functional group	Life history	Photostability	Regeneration	Legacy/new status
Pinus massoniana	Pinaceae	Tree seedling	Perennial	Shade-intolerant	Sprouter	Legacy
Quercus acutissima	Fagaceae	Tree seedling	Perennial	Shade-tolerant	Resprouter	Legacy
Quercus aliena	Fagaceae	Tree seedling	Perennial	Shade-tolerant	Resprouter	Legacy
Dalbergia hupeana	Leguminosae	Tree seedling	Perennial	Shade-tolerant	Resprouter	Legacy
Rhus chinensis	Anacardiaceae	Tree seedling	Perennial	Shade-intolerant	Resprouter	New
Melia azedarach	Meliaceae	Tree seedling	Perennial	Shade-intolerant	Sprouter	New
Morus alba	Moraceae	Tree seedling	Perennial	Shade-tolerant	Sprouter	Legacy
Sapium sebiferum	Euphorbiaceae	Tree seedling	Perennial	Shade-intolerant	Sprouter	New
Diospyros lotus	Ebenaceae	Tree seedling	Perennial	Shade-tolerant	Sprouter	Legacy
Populus davidiana	Salicaceae	Tree seedling	Perennial	Shade-tolerant	Sprouter	Legacy
Vitex negundo	Verbenaceae	Shrub	Perennial	Shade-tolerant	Resprouter	Legacy
Lindera glauca	Lauraceae	Shrub	Perennial	Shade-tolerant	Resprouter	Legacy
Symplocos chinensis	Symplocaceae	Shrub	Perennial	Shade-tolerant	Resprouter	Legacy
Camellia oleifera	Theaceae	Shrub	Perennial	Shade-tolerant	Resprouter	Legacy
Glochidion puberum	Euphorbiaceae	Shrub	Perennial	Shade-tolerant	Sprouter	Legacy
Lespedeza bicolor	Leguminosae	Shrub	Perennial	Shade-tolerant	Resprouter	Legacy
Zanthoxylum schinifolium	Rutaceae	Shrub	Perennial	Shade-intolerant	Sprouter	New
Serissa japonica	Rubiaceae	Shrub	Perennial	Shade-tolerant	Sprouter	Lgacy
Rosa multiflora	Rosaceae	Vine	Perennial	Shade-tolerant	Resprouter	Lgacy
Rosa cymosa	Rosaceae	Vine	Perennial	Shade-tolerant	Resprouter	Lgacy
Smilax china	Liliaceae	Vine	Perennial	Shade-tolerant	Resprouter	Lgacy
Rubus corchorifolius	Rosaceae	Vine	Perennial	Shade-tolerant	Resprouter	Lgacy
Carex rigescens	Cyperaceae	Forb	Perennial	Shade-tolerant	Resprouter	Lgacy
Dryopteris championii	Dryopteridaceae	Fern	Perennial	Shade-tolerant	Resprouter	Lgacy
Lygodium japonicum	Lygodiaceae	Fern	Perennial	Shade-tolerant	Resprouter	Lgacy
Setaria viridis	Gramineae	Graminoid	Annual	Shade-tolerant	Sprouter	New
Phytolacca americana	Phytolaccaceae	Forb	Perennial	Shade-tolerant	Resprouter	New
Conyza canadensis	Compositae	Forb	Annual	Shade-tolerant	Sprouter	New
Oxalis corniculata	Oxalidaceae	Forb	Perennial	Shade-tolerant	Resprouter	New
Cynodon dactylon	Gramineae	Graminoid	Perennial	Shade-tolerant	Resprouter	New

FIGURE S1. The main effects of burning (B) and N addition (N) on the richness (a), cover (b), and density (c) of woody species from June 2014 to October 2016. Two-way ANOVAs were used to examine the effects of burning and N addition on the variables in each sampling date. **p* < .05; ***p* < .01; ****p* < .001.

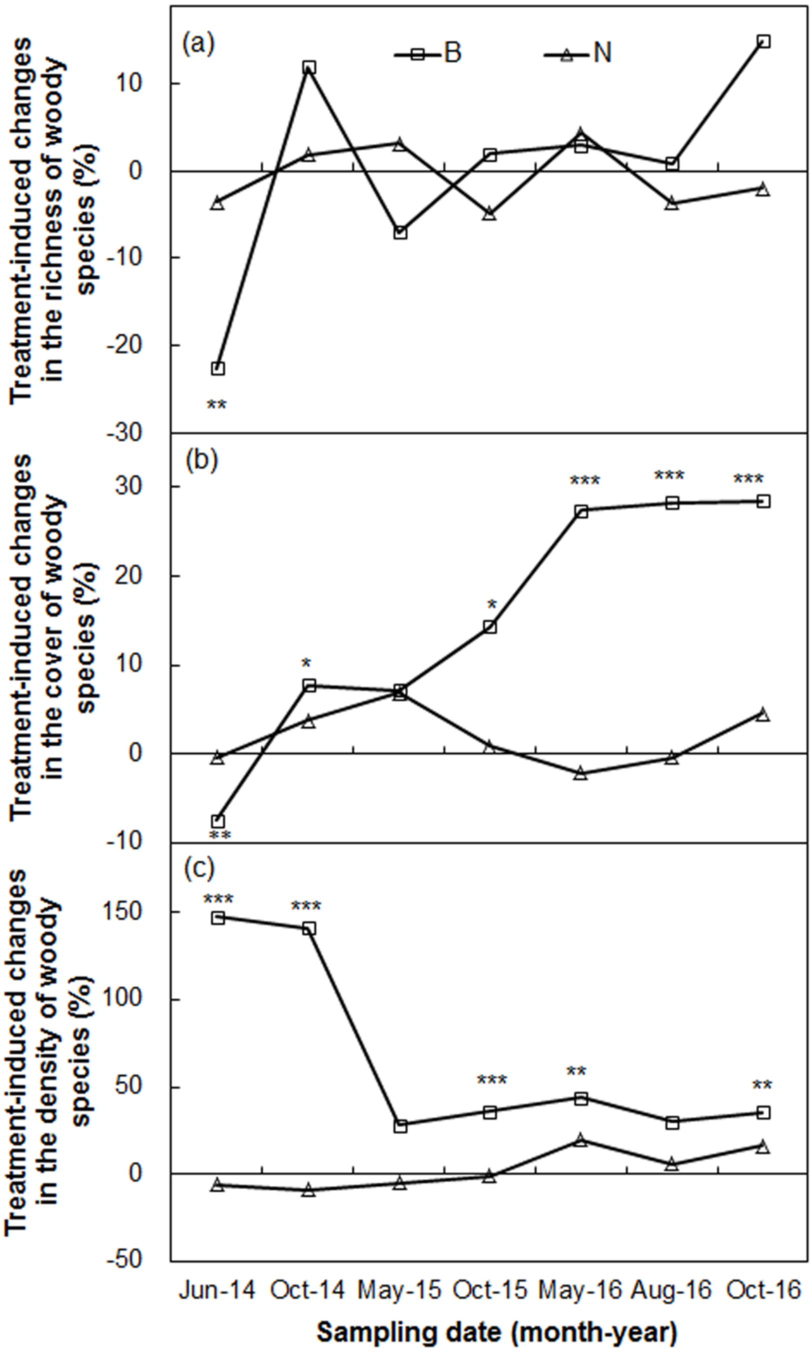

FIGURE S2. The main effects of burning (B) and N addition (N) on herb richness (a), cover (b), and density (c) from June 2014 to October 2016. Two-way ANOVAs were used to examine the effects of burning and N addition on the variables in each sampling date.**p* < .05; ***p* < .01; ****p* < .001.

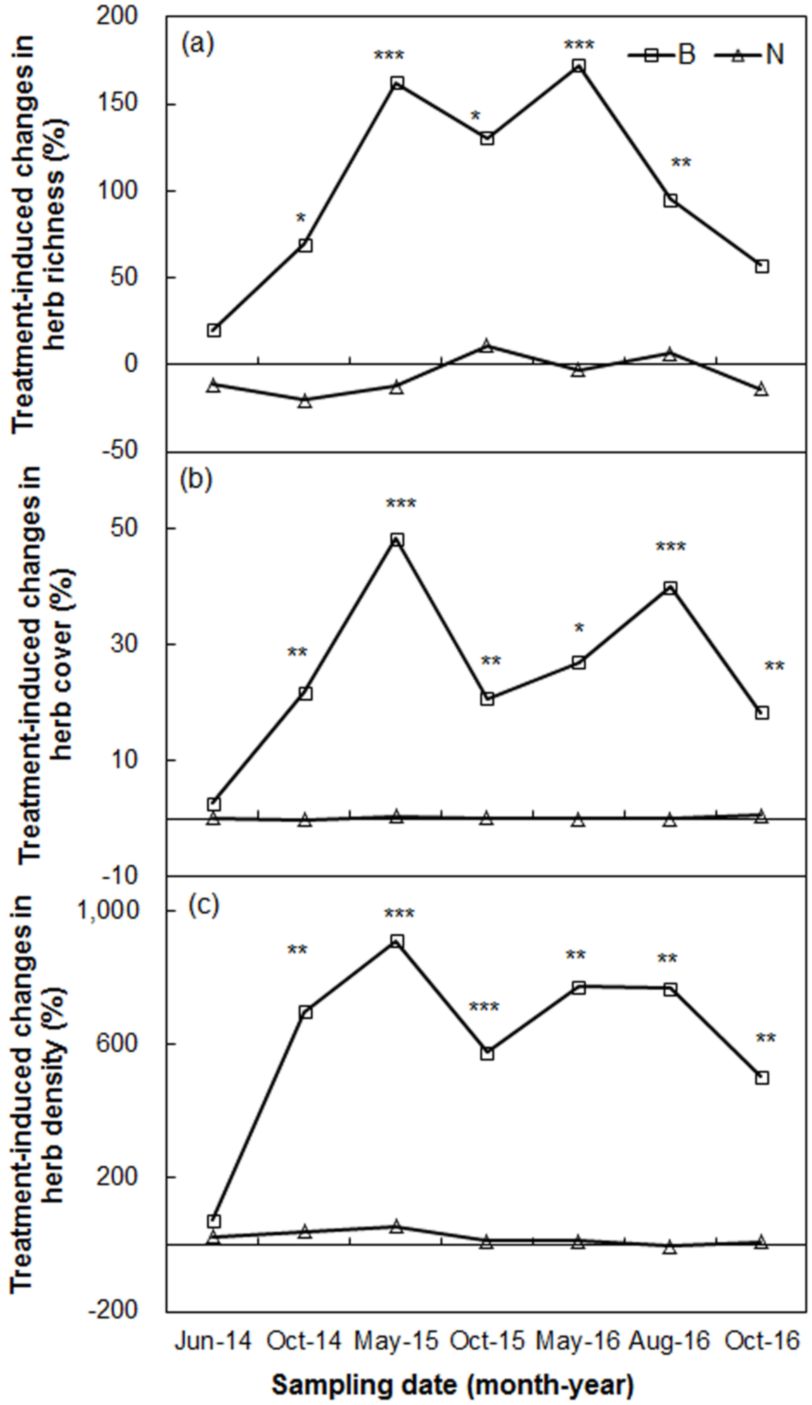

FIGURE S3. Effects of burning and N addition on fine root biomass from 2014 to 2016 (Mean ± SE). C: control; B: burning; N: N addition; BN: burning plus N addition.

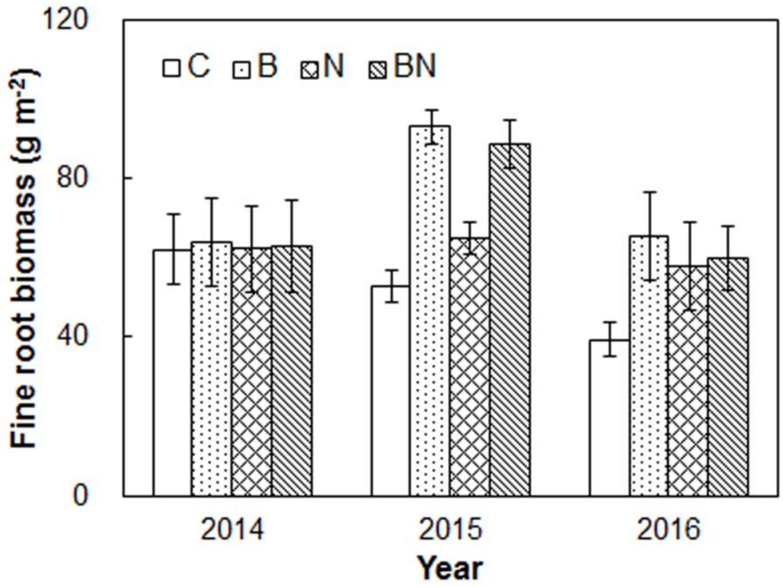

FIGURE S4. Effects of burning and N addition on canopy tree density from 2014 to 2016 (Mean ± SE). C: control; B: burning; N: N addition; BN: burning plus N addition.

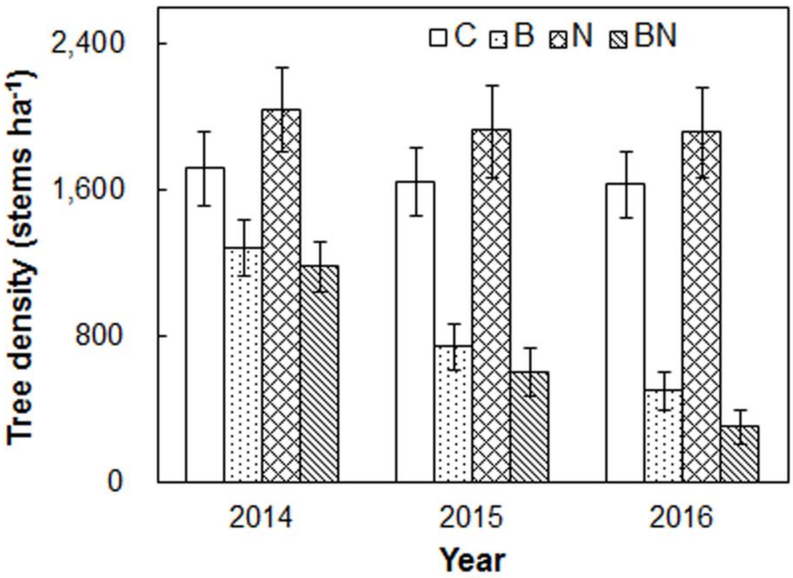

FIGURE S5. Relationship between the cover and height of woody species. Each data point represents the mean value in one quadrat.

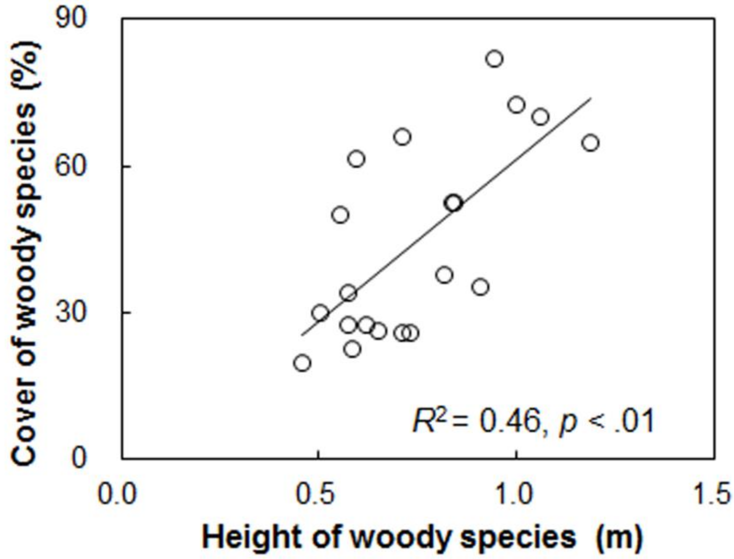

FIGURE S6. Effects of burning and N addition on the density of *C. rigescens* from June 2014 to October 2016 (Mean ± SE). C: control; B: burning; N: N addition; BN: burning plus N addition.

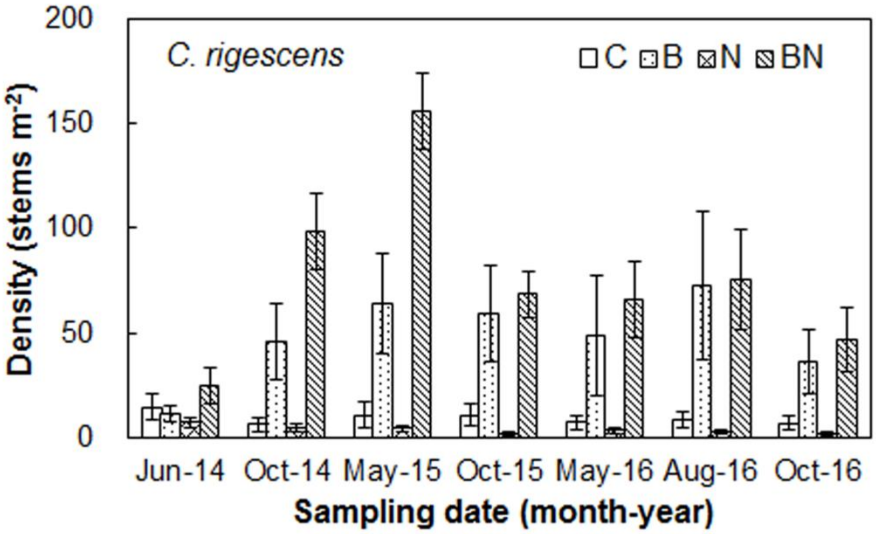

FIGURE S7. Effects of burning and N addition on leaf N content in *V. negundo*, *L. glauca*, and *S. chinensis* in August 2015(Mean ± SE). C: control; B: burning; N: N addition; BN: burning plus N addition.

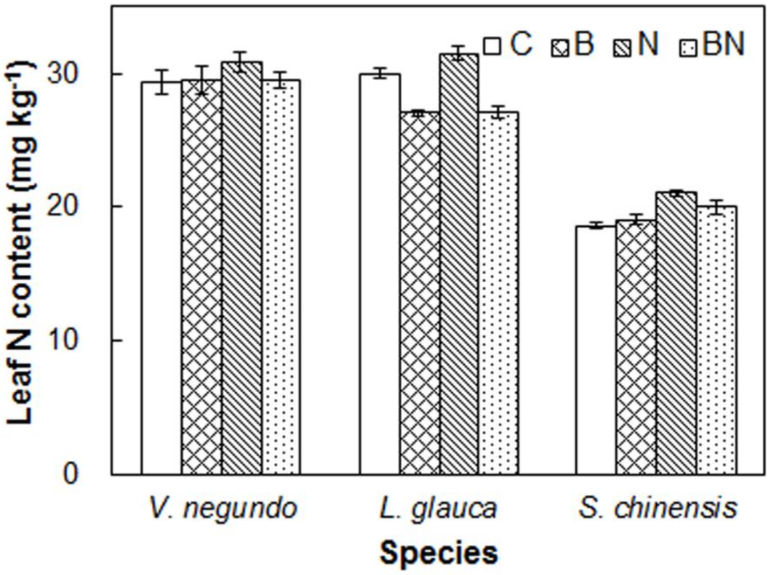

Supplement: Supplementary file 1 [file ECE3-8-8135-s001.pdf]
